# The implementation and utility of clinical exome sequencing in a South African infant cohort

**DOI:** 10.3389/fgene.2023.1277948

**Published:** 2023-11-09

**Authors:** L. Campbell, J. Fredericks, K. Mathivha, P. Moshesh, A. Coovadia, P. Chirwa, B. Dillon, A. Ghoor, D. Lawrence, L. Nair, N. Mabaso, D. Mokwele, M. Novellie, A. Krause, N. Carstens

**Affiliations:** ^1^ Division of Human Genetics, National Health Laboratory Service and School of Pathology, Faculty of Health Sciences, University of the Witwatersrand, Johannesburg, South Africa; ^2^ Department of Paediatrics and Child Health, School of Clinical Medicine, Rahima Moosa Mother and Child Hospital, Faculty of Health Sciences, University of the Witwatersrand, Johannesburg, South Africa; ^3^ Department of Paediatrics and Child Health, School of Clinical Medicine, Nelson Mandela Children’s Hospital, Faculty of Health Sciences, University of the Witwatersrand, Johannesburg, South Africa; ^4^ Nelson Mandela Children’s Hospital, Johannesburg, South Africa; ^5^ Genomics Platform, South African Medical Research Council, Cape Town, South Africa

**Keywords:** clinical-exome, infant, diagnostic, South Africa, LMIC, implementation

## Abstract

Genetic disorders are significant contributors to infant hospitalization and mortality globally. The early diagnosis of these conditions in infants remains a considerable challenge. Clinical exome sequencing (CES) has shown to be a successful tool for the early diagnosis of genetic conditions, however, its utility in African infant populations has not been investigated. The impact of the under-representation of African genomic data, the cost of testing, and genomic workforce shortages, need to be investigated and evidence-based implementation strategies accounting for locally available genetics expertise and diagnostic infrastructure need to be developed. We evaluated the diagnostic utility of singleton CES in a cohort of 32 ill, South African infants from two State hospitals in Johannesburg, South Africa. We analysed the data using a series of filtering approaches, including a curated virtual gene panel consisting of genes implicated in neonatal-and early childhood-onset conditions and genes with known founder and common variants in African populations. We reported a diagnostic yield of 22% and identified seven pathogenic variants in the NPHS1, COL2A1, OCRL, SHOC2, TPRV4, MTM1 and STAC3 genes. This study demonstrates the utility value of CES in the South African State healthcare setting, providing a diagnosis to patients who would otherwise not receive one and allowing for directed management. We anticipate an increase in the diagnostic yield of our workflow with further refinement of the study inclusion criteria. This study highlights important considerations for the implementation of genomic medicine in under-resourced settings and in under-represented African populations where variant interpretation remains a challenge.

## 1 Introduction

Genetic conditions are significant contributors to infant mortality, morbidity, and hospitalisations globally (Kingsmore et al., 2020). Despite advances in diagnostics with next-generation sequencing (NGS) and microarrays, it remains challenging to make diagnoses in infant populations. Infants often present with atypical or non-specific disease symptoms; many genetic disease phenotypes cannot be distinguished during the neonatal period; and disease progression may often be very rapid, making the identification and diagnosis of genetic conditions difficult (Wilkinson et al., 2016; van Diemen et al., 2017; French et al., 2019).

Clinical and whole exome sequencing (CES and WES), have been widely investigated for their use in diagnosing ill infants, with many studies evidencing their benefit (Petrikin et al., 2015; Smith et al., 2015; Willig et al., 2015; Stark et al., 2016; Meng et al., 2017; van Diemen et al., 2017; Farnaes et al., 2018; French et al., 2019; Kingsmore et al., 2019; Lunke et al., 2020; Wang et al., 2020). While WES targets all protein-coding regions of the genome, CES targets select genes with known disease associations and their flanking splice regions, and has been shown to provide an early, definitive diagnosis, preventing a diagnostic odyssey, and allowing for targeted clinical management (Smith et al., 2015). Virtual panels examining a subset of these clinically relevant genes from WES/CES data can be utilized to minimise the initial time and cost of analysis while offering the potential of comprehensive analysis of additional genes at a later stage with no additional laboratory costs, a key cost consideration for resource-constrained settings. Previous studies suggest that earlier diagnoses promote improved patient care and outcomes by facilitating changes in medical treatment, the early introduction of targeted therapies, increased surveillance, the appropriate initiation of comfort care, the reduction of costly, repeat, and sometimes invasive investigations, and the instigation of cascade testing for family members and counselling for reproductive planning–all measurable utility for the use of diagnostic testing (Rabbani et al., 2012; Splinter et al., 2018; Wright et al., 2018; Lunke et al., 2020).

Despite evidence from numerous research studies illustrating the benefits of CES for the diagnosis and management of ill infants, CES is not accessible in many parts of the world, particularly in low- and middle-income countries (LMICs). In many LMICs, CES is only offered in limited research settings (or not at all) creating larger health disparities in regions where access to primary healthcare and diagnostic services are already poor. This is true across the African continent, with few countries having established genetic services (Kamp et al., 2021). In South Africa, only three of the nine provinces offer genetic services in the State healthcare system, which services more than 80% of the population (Kromberg et al., 2013a; Stats SA, 2022). With limited access to genetic services, many affected by genetic conditions go undiagnosed and untreated. The lack of funding and resources for CES implementation is widely acknowledged by stakeholders, particularly shortages in infrastructure and trained genetics professionals (Kamp et al., 2021; Lumaka et al., 2022). The implementation of CES in the South African State healthcare system needs to be informed by international guidelines and standards, but requires optimisation in the local context to accommodate the scarcity of resources and the limited bioinformatics and genomics capacity (Kamp et al., 2021). The implementation of CES is further complicated by challenges in genetic data interpretation due to the underrepresentation of African populations in genomic databases and literature, African genetic diversity, and the lack of disease registries to document genetic disease prevalence (Kromberg et al., 2013b; Baynam et al., 2020; Lumaka et al., 2022). Despite these challenges, CES may offer significant improvements in the clinical management of vulnerable, underserved South African populations, including ill infants in the neonatal intensive care unit (NICU). Without a genetic diagnosis, infants with genetic conditions are constrained by less accurate risk assessments, prognoses, and specialist referrals; minimal assistance from support organisations and government welfare; and no access to emerging therapies and clinical trials, widening the existing healthcare disparities African populations face in accessing personalised healthcare.

With the shift in focus of genomic medicine to implementation science and translation into clinical practice, studies are needed to determine the benefits and challenges faced in implementing CES in real-world settings (Kingsmore and Cole, 2022). There are many barriers to the implementation of genomic medicine in LMICs which need to be investigated and addressed for genomic medicine and CES to be successfully integrated into global healthcare systems. To meet the World Health Organisation’s sustainable development goal to reduce neonatal and children under-5 mortality, the implementation of adequate genetic services to address the burden of genetic disease, often overshadowed and masked by infectious and communicable disease, is necessary in LMICs. Implementation should address the various barriers faced, including limited infrastructure and technology, shortages in the genetics workforce, poor genomic literacy amongst healthcare providers, poor literacy and education amongst the general population, language barriers and the lack of standardised genomics terminology in local languages, cultural and societal nuances around family structure, cultural beliefs around disease causality, and paucity of information around genetic disease burden in African populations (Kamp et al., 2021). These challenges will vary across LMICs due to population, economic, political, and social diversity, rendering a one-size-fits all implementation approach inappropriate for most settings and making investigations into unique, country-specific challenges necessary (Tiffin, 2014; Kamp et al., 2021; Lumaka et al., 2022).

We performed a scoping study to evaluate the diagnostic utility of singleton CES in a cohort of ill infants suspected of having a genetic condition in the South African State healthcare system through a series of three virtual gene panels. This study provides insight into the implementation of CES for ill infants in an under-resourced LMIC setting, where access to genetic services is limited, the burden of genetic disease is unknown and the interpretation of genomic data remains a considerable challenge.

## 2 Methods

**FIGURE 1:**
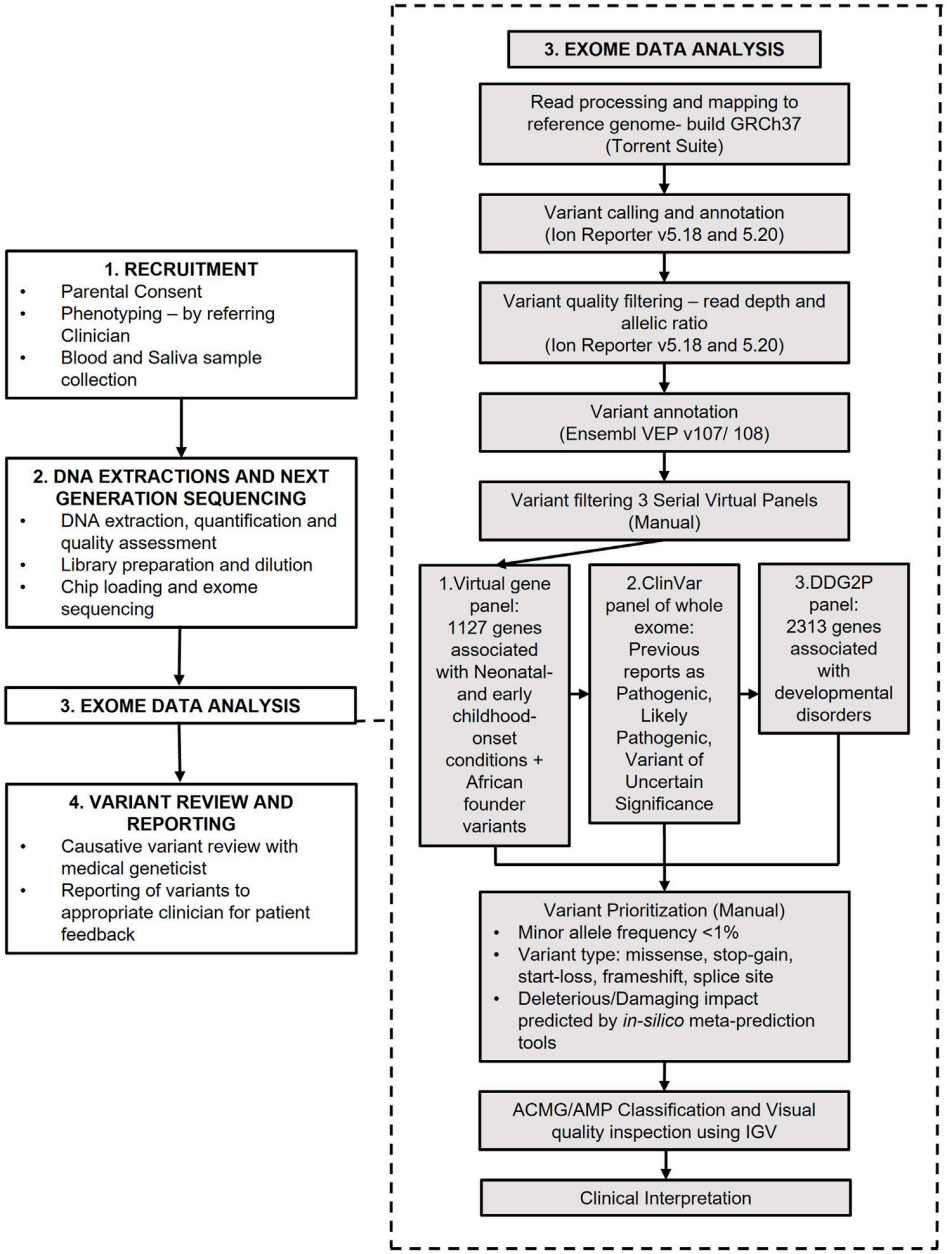
Study overview and analysis workflow.

The study workflow is summarised in Figure 1.

### 2.1 Recruitment

A cohort of thirty-two ill infants, suspected of having a genetic condition, were recruited from two State hospitals in Johannesburg, South Africa: Rahima Moosa Mother and Child Hospital (RMMCH) in Coronationville, and Nelson Mandela Children’s Hospital (NMCH) in Parktown. These hospitals are regional and quaternary hospitals respectively and serve mostly State patients. Participants were referred for genetic testing either directly by neonatologists and paediatricians without consultation with medical geneticists, or by medical geneticists at call out consultations or referral. Clinicians were provided a list of recruitment criteria, highlighting a broad range of phenotypic features that may be suggestive of a genetic cause for an ill infant’s condition. Infants were considered eligible for recruitment if they presented with any of the following: multiple congenital anomalies, a concern for their neurological status, metabolic abnormalities of uncertain cause, dysmorphic features or if they had abnormal growth parameters. Infants were excluded if they had significant teratogen exposure during pregnancy, experienced birthing trauma or asphyxia, or had a diagnosed infection that could be the primary cause of illness. Premature patients were considered eligible for inclusion if their clinical features were not primarily explained by prematurity.

Parents and guardians provided informed consent on behalf of infants for participation in the study after the study was explained to them by a principal investigator or genetic counsellor. A blood sample was collected for DNA extraction and exome testing from each participant. A saliva sample from available parents was banked at consent, however, this study employed exome sequencing of probands only.

Phenotypic information regarding the condition and medical history of the participants was provided by the referring clinician. Clinicians were provided a short format phenotype collection rubric to assist with this as a Redcap form (Supplementary Table S1) (Harris et al., 2009; Harris et al., 2019), and requested to provide any information they believed was relevant to a possible genetic diagnosis.

### 2.2 DNA extraction and next-generation sequencing

DNA extraction was performed on the stored participant blood samples using a modified salting-out method (Miller et al., 1988). DNA quality and quantity was assessed using the NanoDrop 2000 (Thermo Fisher Scientific, United States), gel electrophoresis and the Qubit 4.0 system (Thermo Fisher Scientific, United States). Library preparation was performed manually using the Ion Ampliseq Exome RDY library preparation kit, as per the manufacturer’s instruction. DNA libraries were quantified and sized using the TapeStation High Sensitivity D5000 ScreenTape assay (Agilent Technologies, Germany). Diluted libraries were loaded onto Ion 540 sequencing chips for exome sequencing using the automated Ion Chef instrument (Thermo Fisher Scientific, United States). Loaded chips were sequenced in-house on an Ion GeneStudio S5 sequencer (Thermo Fisher Scientific, United States). The laboratory processing time for four samples was approximately 5 days.

### 2.3 Exome data analysis

Read alignment to the GRCh37 human reference genome was performed on the Ion Torrent Suite software (Thermo Fisher Scientific, United States). Variant calling was subsequently performed using Ion Reporter software (Thermo Fisher Scientific, United States). Variants were annotated using the Ensembl Variant Effect Predictor (VEP), versions 107 and 108 (McLaren et al., 2016).

Variants were filtered and prioritised for manual curation using three sequential filtering approaches, summarised in Figure 1. The first variant filtering approach retained variants in a virtual gene panel consisting of 1,127 genes, curated for their association with neonatal- and early-childhood-onset conditions (Ceyhan-Birsoy et al., 2017; Milko et al., 2019) and genes with known founder variants and common variants in African populations (Krause et al., 2018) (NICU/African) (Supplementary Table S2). If no causative variant was identified, the second variant filtering approach, retaining variants with previous pathogenic, likely pathogenic and variant of uncertain significance (VUS) classifications on ClinVar, including variants with conflicting interpretations, was employed (Landrum et al., 2018). The third filtering strategy, employed if no causative variant was identified from the first two strategies, retained variants in 2,313 genes on the Deciphering Developmental Disorders Genotype to Phenotype (DDG2P) gene panel (Thormann et al., 2019). Filtered variants were then prioritised for manual curation based on the type of variant, population minor allele frequencies, previous pathogenic and likely pathogenic interpretations on ClinVar and predicted deleterious impacts on protein function from *in silico* prediction tools CADD, REVEL, BayesDel and MetaRNN (Ioannidis et al., 2016; Feng, 2017; Rentzsch et al., 2019; Li et al., 2022). Variants were visually inspected to determine quality using Integrated Genomics Viewer software (IGV) (Robinson et al., 2011).

Prioritised variants were classified according to the American College of Medical Genetics and Genomics and the Association for Molecular Pathology (ACMG/AMP) guidelines to determine their pathogenicity (Richards et al., 2015). Filtering, prioritization, manual curation and classification took approximately 6 h to perform per filtering strategy per sample. Pathogenic and likely pathogenic variants were then reviewed by a multidisciplinary team to determine if the diagnosis was appropriate for the participant. Findings were reported in a research report and returned to the referring clinicians and participants. VUSs and incidental findings were not reported in this study. Positive molecular findings were returned to parents by a genetic counsellor or medical geneticist, and appropriate downstream medical intervention and referrals initiated where necessary. Parents of participants with negative findings were invited for further consultation with a medical geneticist to discuss the implications of the negative result and determine an appropriate clinical management route going forward.

## 3 Results

### 3.1 Cohort characteristics

Thirty-two infants under the age of two, with a suspicion of a genetic condition were recruited for CES between May 2021 and December 2022 (Table 1). A single participant was enrolled and excluded as the participant had received a blood transfusion and demised before an appropriate amount of time had passed for a blood sample to be drawn. Most participants were referred for genetic testing directly by their treating neonatologist or paediatrician (23) and were not formally assessed by a medical geneticist prior to recruitment. An equal proportion of male and female participants were recruited. Most participants were of African ancestry (26). Only two participants, NE025 and NE035, were born to self-reported consanguineous parents. The most frequent indications for CES were multiple congenital anomalies (9), cardiac defects and abnormalities (6), general dysmorphism (3) and neuromuscular abnormalities (3). No participants had abnormalities detected prenatally on ultrasound or had prenatal genetic investigations, however, fetal anomaly scans are infrequently performed as standard obstetric care in the State healthcare system.

**TABLE 1:** Demographics and clinical characteristics of the patient cohort referred for clinical exome sequencing.

Characteristic		Number of individuals	Percentage of cohort (%)	Number of positive diagnoses	Percentage of positive diagnoses (%)
Total number of participants		32	—	7	21.9
Hospital recruitment site	RMMCH	17	53.1	6	35.3
	NMCH	15	46.9	1	6.7
Sex	Female	16	50.0	3	18.8
	Male	16	50.0	4	25.0
Referring clinician	Neonatologist/Paediatrician	23	71.9	4	17.4
	Geneticist	9	28.1	3	33.3
Ancestry	African	26	81.3	5	19.2
	European	1	3.1	—	—
	Mixed	1	3.1	1	100.0
	Other	4	12.5	1	25.0
Consanguinity	Consanguineous parents	2	6.3	—	0.0
	Non-consanguineous parents	30	93.8	7	−23.3
Indication for genetic testing	Neuromuscular	3	9.4	2	66.7
	Skeletal	2	6.3	2	100.0
	Cardiac	6	18.8	—	—
	Renal	2	6.3	1	50.0
	Multiple congenital anomalies	9	28.1	1	11.1
	Hepatic	2	6.3	—	—
	Developmental	2	6.3	1	50.0
	Abnormal growth	1	3.1	—	—
	Dysmorphic	3	9.4	—	—
	Neurological	1	3.1	—	—
	Gastrointestinal	1	3.1	—	—

Abbreviations: RMMCH, Rahima Moosa Mother and Child Hospital; NMCH, Nelson Mandela Children’s Hospital.

### 3.2 Genetic diagnoses from singleton CES

Singleton CES was performed on all participants with an average coverage of 134%X and 90% uniformity. A molecular diagnosis was confirmed for 7 of the 32 recruited participants, resulting in a diagnostic yield of 22% (Table 2). Five of the identified causative variants were in genes in our NICU/African virtual panel.

**TABLE 2:** Positive infant diagnoses through clinical exome sequencing using three filtering strategies.

Study ID	Hospital	Referring clinician type	Sex	Ancestry	Indication for testing	Phenotypic features	Variant HGVS; gene OMIM number	Variant type	Filtering strategy	ACMG/AMP classification and codes	ClinVar accession ID and classification	Variant inheritance, variant coverage	Diagnosed condition
NE001	RMMCH	Neonatologist/Paediatrician	M	African	Neuromuscular	Myopathic facies, generalised hypotonia, reduced tendon reflexes, low set, posteriorly rotated ears, high arched palate, cryptorchidism, wide intermammillary distance, genu recurvatum, rocker bottom foot, single transverse palmar crease	*STAC3*:c.851G > C (p.Trp284Ser) 615521	Missense	NICU/African Panel	Pathogenic—PM3_strong, PS3, PP3, PP5_supporting	VCV000088744.39 2 star Pathogenic	Autosomal recessive, 124X	STAC3 (Bailey-Bloch) myopathy
NE021	RMMCH	Neonatologist/Paediatrician	M	Other - Pakistani	Neuromuscular	General hypotonia, myopathic facies, frog-like posture, areflexia, low power, breathing difficulties, open anterior and posterior fontanelle, generalised hypertrichosis, retrognathia, narrow chest, hypoplastic scrotum, cryptorchidism	*MTM1*:c.664C > T (p.Arg222Ter) 300415	Stop gain	NICU/African Panel	Pathogenic—PVS1, PM2_supporting, PP5_supporting	VCV000158994.14 2 star Pathogenic	X-linked recessive, 47X	X-linked myotubular myopathy
NE022	NMCH	Geneticist	F	African	Skeletal	Mild rhizomelic shortening, restricted elbow extension and hip abduction, truncal hypotonia, short sternal prominence, pectus carinatum, short and narrow chest, Harrison’s sulcus, flared ribs, protuberant abdomen with palpable soft liver and spleen, exaggerated lumbar lordosis, relative macrocephaly, flat nasal bridge, prominent forehead, epicanthal folds, infraorbital creases, grey sclera, malar hypoplasia, short neck	*COL2A1*:c.3589G > A (p.Gly1197Ser) 120140	Missense	NICU/African Panel	Likely Pathogenic—PM5, PM1, PM2_supporting, PP3, PP2, PP5_supporting	VCV000017361.21 2 star Pathogenic/Likely Pathogenic	Autosomal dominant, 85X	COL2A1 collagen disorder
NE026	RMMCH	Neonatologist/Paediatrician	M	African	Developmental	Neonatal seizures, hypocalcaemia, failure to thrive, alopecia, sparse eyebrows, frontal bossing, scaphocephaly, malar flattening, flat nasal bridge, retromicrognathia, mild abdomen distention, scrotal dysplasia, mild hypotonia with normal power, dry skin, feeding difficulties due to fragile teeth and bleeding gums	*SHOC2*:c.4A > G (p.Ser2Gly) 602775	Missense	ClinVar Filter	Pathogenic -—PS2_very strong, PS4, PS3_moderate, PM2_supporting, PP2, PP5_supporting	VCV000006821.64 3 star Pathogenic	Autosomal dominant, 34X	Noonan-like syndrome with loose anagen hair
NE029	RMMCH	Geneticist	M	Mixed	Multiple congenital anomalies	Cataracts, blocked tear ducts, bilateral eye opacity, bilateral nystagmus, retrognathia, open anterior fontanelle without craniotabes, redundant skin at shoulder joints, brachydactyly, tapered fingers, hyperlaxity of finger joints, short stature, global hypotonia with reduced reflexes and power, head lag, osteomalacia on x-ray, proteinuria, stagnating weight	*OCRL*:c.1621C > T (p.Arg541Ter) 300535	Stop gain	NICU/African Panel	Pathogenic - PVS1_strong, PM2_supporting, PP5_supporting	VCV000521093.9 2 star Pathogenic	X-linked recessive, 16X	Lowe syndrome
NE033	RMMCH	Geneticist	F	African	Renal	Congenital nephrotic syndrome, left ventricular hypertrophy, effusion of right ventricle, peripheral pulmonary stenosis, anasarca, proteinuria, electrolyte abnormalities, hypothyroidism	*NPHS1*:c.1379G>A (p.Arg460Gln) 602716	Missense	NICU/African Panel	Likely Pathogenic - PM3_strong, PM1, PP5_supporting	VCV000056438.17 2 star Pathogenic	Autosomal recessive 89X	Congenital nephrotic syndrome
NE034	RMMCH	Neonatologist/Paediatrician	F	African	Skeletal	Metaphyseal widening left femur and midshaft fracture of right femur, hypoplastic left tibia, restricted elbow extension, proximally inserted thumb, tapered fingers bilaterally, fixed hip flexion bilateral hip and knee extension, bilateral talipes equinovarus, hypoplastic nails both feet, prominent occiput, Mongolian blue spot over buttocks and sacral, dimple over medial malleoli, reduced plantar creases	*TRPV4*:c.806G > A (p.Arg269His) 605427	Missense	ClinVar Filter	Pathogenic—PS4, PS3, PM5, PM1, PP5_supporting	VCV000005000.37 2 star Pathogenic	Autosomal dominant, 73X	Spondylo-metaphyseal dysplasia

Abbreviations: RMMCH, Rahima Moosa Mother and Child Hospital; NMCH, Nelson Mandela Children’s Hospital; M, Male; F, female; NICU/African, Neonatal- and early childhood onset and African Founder Variant.

Two participants received a severe myopathy diagnosis. A homozygous *STAC3* variant (c.851G>C; p.Trp284Ser) was identified in participant NE001. This variant has been associated with a diagnosis of *STAC3* myopathy (Horstick et al., 2013; Telegrafi et al., 2017; Waldrop et al., 2017; Zaharieva et al., 2018; Schoonen et al., 2019; Ravenscroft et al., 2021; Saleh et al., 2021). Multiple *in silico* prediction tools predict this variant to be deleterious to STAC3 function, confirmed in zebrafish models, which show a deficiency in skeletal muscle excitation-contraction coupling as a result of this missense mutation (Horstick et al., 2013; Linsley et al., 2017). A hemizygous, nonsense *MTM1* variant (c.664C>T; p.Arg222Ter), affecting the functional myotubularin phosphatase domain of the MTM1 protein, was identified in participant NE021. This variant has been associated with severe X-linked myotubular myopathy (Laporte et al., 1997; Tanner et al., 1999; Biancalana et al., 2003; Longo et al., 2016). Both diagnosed myopathies had severe disease presentations, resulting in the demise of both infants within a week of life. Parents of these infants were offered prenatal testing for future pregnancies.

A pathogenic, heterozygous missense variant in *COL2A1* (c.3589G > A; p.Gly1197Ser) was identified in participant NE022. This variant occurs in a predicted missense constrained gene and has predicted deleterious effects on type II collagen stability and structure (Beck et al., 2000). Other amino acid substitutions at the same position, p.Gly1197Ala and p.Gly1197Arg, have previously been reported as pathogenic. Mutations in the *COL2A1* gene have been associated with a spectrum of overlapping skeletal dysplasias, therefore, this participant received a broad diagnosis of a *COL2A1* collagen disorder (Cole et al., 1993; Nishimura et al., 2005; Terhal et al., 2015). Further assessments are needed to refine this diagnosis. This participant will need to be monitored for eye abnormalities, cervical instability and have their respiratory function assessed by the appropriate medical specialties.

Participant NE026 was diagnosed with Noonan-like syndrome with loose anagen hair caused by a heterozygous *SHOC2* variant (c.4A > G, p.Ser2Gly). This variant, carrying a three-star ClinVar classification, has been shown to alter MAPK activation in *in vitro* studies and animal models (Cordeddu et al., 2009; Hoban et al., 2012; Gripp et al., 2013; Gargano et al., 2014; Choi et al., 2015). This participant will be referred for neurodevelopmental and cardiac assessments, as cardiac defects and psychomotor delay are frequently observed in this condition.

A hemizygous, nonsense *OCRL* variant (c.1621C > T, p.Arg541Ter) was identified in participant NE029, associated with X-linked recessive Lowe (Oculo-cerebro-renal) syndrome. This specific premature termination variant has been observed in other Lowe syndrome patients with similar clinical presentations (Hichri et al., 2011; Yang et al., 2014; Recker et al., 2015). This participant will require ophthalmology treatment for cataracts and glaucoma, as well as electrolyte monitoring for renal tubular acidosis. The mother of Participant NE029 received carrier screening by Sanger sequencing, confirming that she was not a carrier for the identified pathogenic variant.

Participant NE033 received a diagnosis of severe congenital nephrotic syndrome (Finnish type), harbouring a homozygous variant in the *NPHS1* gene (c.1379G > A, p.Arg460Gln). This variant has been reported in a homozygous state in multiple congenital nephrotic syndrome cases (Beltcheva et al., 2001; Schoeb et al., 2010; Warejko et al., 2018; Mann et al., 2019). Participant NE033 presented with end stage renal disease and was unable to access renal replacement therapy in South Africa. Due to a poor prognosis and limited available medical intervention, this family received counselling on comfort care.

A heterozygous missense variant in *TRPV4* (c.806G > A, p.Arg269His) was identified in participant NE034. This variant has been extensively reported in the literature for its association with autosomal dominant neuromuscular disease and skeletal dysplasia (Auer-Grumbach et al., 2010; Deng et al., 2010; Landoure et al., 2010; Zimon et al., 2010; Echaniz-Laguna et al., 2014; Louis et al., 2014; Biasini et al., 2016; Fleming and Quan, 2016; Jedrzejowska et al., 2019). Multiple functional studies support this variant’s gain of function role in increasing calcium channel activity, resulting in cell death and axonal degeneration (Deng et al., 2010; Landoure et al., 2010; Fecto et al., 2011; Klein et al., 2011; Takahashi et al., 2014). Participant NE034 presented clinically with only the skeletal features of *TRPV4*-associated disease and received a diagnosis of spondylometaphyseal dysplasia. As the neuropathic symptoms of this disease have been reported to occur after the neonatal period, this participant will need to be monitored for the development of these symptoms (Fleming and Quan, 2016). This participant will require physical therapy to maintain lower limb function. Pulmonary function and cervical spine stability will also be monitored in this participant.

## 4 Discussion

This study generated valuable insights into the practicalities and utility of implementing CES in a NICU setting in South Africa. As the developed world moves toward rapid testing for ill infants, challenges in the implementation of genomic medicine in African and LMICs exacerbates existing healthcare disparities due to the slow uptake of these newer technologies, particularly in Sub-Saharan Africa, where most NGS testing, even in the research setting, is performed outside of the African continent (Baine-Savanhu et al., 2023). These challenges require careful consideration for the implementation of cutting-edge genomic services and for health equity to be achieved (Kingsmore and Cole, 2022).

We identified a genetic cause in seven ill infants with a range of clinical disease presentations, achieving a minimum diagnostic yield of 22%. Many studies globally have assessed the utility of CES in infant populations, showing impressive diagnostic yields of up to 70% (Clark et al., 2018), however, there is an absence of African individuals in these studies and low representation from LMICs. Patients of African ancestry are less likely to receive a diagnosis using current analytical strategies, as shown in the Deciphering Developmental Disorders study, creating a significant healthcare disparity for these populations (Wright et al., 2023). A tailored implementation strategy is needed for successful uptake of CES in Africa and in LMICs.

Comprehensive analysis strategies offer a higher probability of identifying a causative variant, however, these strategies are more time and labour intensive and have higher VUS rates (Stark and Ellard, 2022). A curated virtual panel of carefully selected genes, as demonstrated in this study, is an effective first approach for exome data analysis. Five of the seven causative variants identified in this study were in genes in our custom NICU/African panel, illustrating its utility for infant cohorts. The simplification of analysis pipelines and strategies in LMIC settings is necessary to deliver a result in a timely manner and with a low dependence on computational infrastructure and bioinformatics capacity. More comprehensive analysis pipelines, such as whole exome analysis, and the investigation of CNVs and structural variants could be deployed next for the remaining undiagnosed participants (Splinter et al., 2018). Undiagnosed participants may also benefit from reanalysis to incorporate updates to the virtual gene panel, new variant information, novel gene-disease associations, updated phenotypic data and bioinformatic advancements (Schobers et al., 2022).

The implementation of CES across the African continent and in LMICs is significantly under-investigated and many of the challenges associated with CES implementation into genetic service are exacerbated in an African setting. The significant barriers to the implementation of genomic medicine include a lack of infrastructure and support for clinical translation, the scarcity of information regarding African genetic epidemiology, poor genomic literacy among healthcare workers, few formally trained genetics professionals, a lack of investment by governments and international funders, the low cost effectiveness in establishing genetic services, and the fear of widening the existing disparities by only benefiting those who can afford access to these technologies (Jongeneel et al., 2022). These challenges, many seen in this study, need to be addressed when planning implementation strategies for CES in our context.

More than 70% of our infant cohort was referred for genetic testing directly by a neonatologist or paediatrician. Appropriate phenotyping and a predictive clinical diagnosis from a medical geneticist can direct CES data interpretation and contribute to a higher diagnostic yield (Trujillano et al., 2017; Gubbels et al., 2020). The limited formally trained genetics workforce in South Africa makes input from trained geneticists prior to genetic testing a virtually impossible task (Kromberg et al., 2013a). The engagement and upskilling of primary care medical specialities is necessary for CES implementation in under-resourced settings, to allow the few medical geneticists and genetic counsellors to be most efficiently utilised (Dragojlovic et al., 2020; Chou et al., 2021).

The lack of representation of African and Sub-Saharan African populations in publicly available databases and limited understanding of African genetic disease epidemiology makes CES data analysis and interpretation complex. A representative reference genome which considers African population diversity and disease epidemiology is essential in identifying disease-causing variants in a timely manner. A better understanding of genetic disease in African populations is key to improved management and service of our populations. This study allowed us to investigate genetic disease epidemiology in an underrepresented African population. The variant identified in participant NE001, diagnosed with *STAC3* myopathy, has the highest carrier rate in the Genome Aggregation Database (GnomAD, accessed June 2023) in individuals of African/African American ancestry (Zaharieva et al., 2018; Gromand et al., 2022). Unpublished research from the South African National Health Laboratory Service revealed a frequency of 20% for this variant in a homozygous or compound heterozygous state in *SMN1* negative African patients with myopathic phenotypes (Mhlongu et al., 2022). Despite its prevalence in African-ancestry patients, *STAC3* testing has only been recognized as a common myopathy and introduced into diagnostic service in the South African State system within the past year.

Singleton CES was utilised in this study to elucidate the potential cause of disease in our infant cohort. Despite its recognised diagnostic superiority, trio-sequencing is costly to consider in LMICs, where limited financial resources must effectively serve large populations {Baine-Savanhu et al., 2023 #416} (Clark et al., 2018; Kingsmore et al., 2019). Trio-sequencing is thus not affordable at present in LMICs and the benefit versus cost requires further investigation. Complex family dynamics and social issues are other key considerations for African populations. In this cohort, only 31% of participants had both parents available at enrolment due to a variety of social factors, including: admission at hospitals far away from their residence with no access to transportation; the inability to get time off work for hospital visits; some participants were African immigrants who were not in South Africa as a complete family unit; and many participants were born to single-parent households and families. A diagnosis was still achievable in some participants in this study with singleton sequencing, illustrating the effectiveness and utility of this tool as a first-tier strategy, despite difficulties in documenting *de novo* mutations and reoccurrence risk. For effective implementation of genomic services in Africa, these social challenges need to be embraced to prevent the exclusion of patients who would greatly benefit from access to these services.

Approximately 80% of participants did not receive a molecular diagnosis in this study. The workflow offers a starting strategy in the diagnostic trajectory of these participants, who would benefit from further investigations to fill in gaps not addressed in our virtual gene panels. Refinement of the inclusion and exclusion criteria may provide more clarity to referring clinicians as to which infants would most benefit from CES, allowing for better utilisation of the scarce resources for testing. More comprehensive phenotyping may also provide more guidance in participant screening and in variant interpretation for a more cost- and time-effective implementation strategy.

In conclusion, this study yielded a definitive diagnosis for seven families affected by a genetic condition with minimal access to genetic testing in the South African State healthcare system, providing the first evidence of the diagnostic utility of CES for ill infants in an under-resourced NICU setting in Africa. Our experience can be used as a point of departure to develop feasible, local CES implementation strategies, utilising appropriately curated virtual panels to provide clinically actionable findings within an appropriate timeframe. Optimisation of an appropriate gatekeeping strategy that leverages locally available genetics capacity may increase our diagnostic yield and minimise inappropriate testing.

## Data Availability

The data presented in the study are deposited into ClinVar, Organisation ID 508172 and can be accessed at https://www.ncbi.nlm.nih.gov/clinvar/submitters/508172/.
