# Supplementary material for: The implementation and utility of clinical exome sequencing in a South African infant cohort
Source: Front Genet. 2023 Nov 9;14:1277948. doi: 10.3389/fgene.2023.1277948 (PMC10665497; doi:10.3389/fgene.2023.1277948)
Supplement: Supplementary file 1 [file Presentation1.PPTX]

## Slide 1
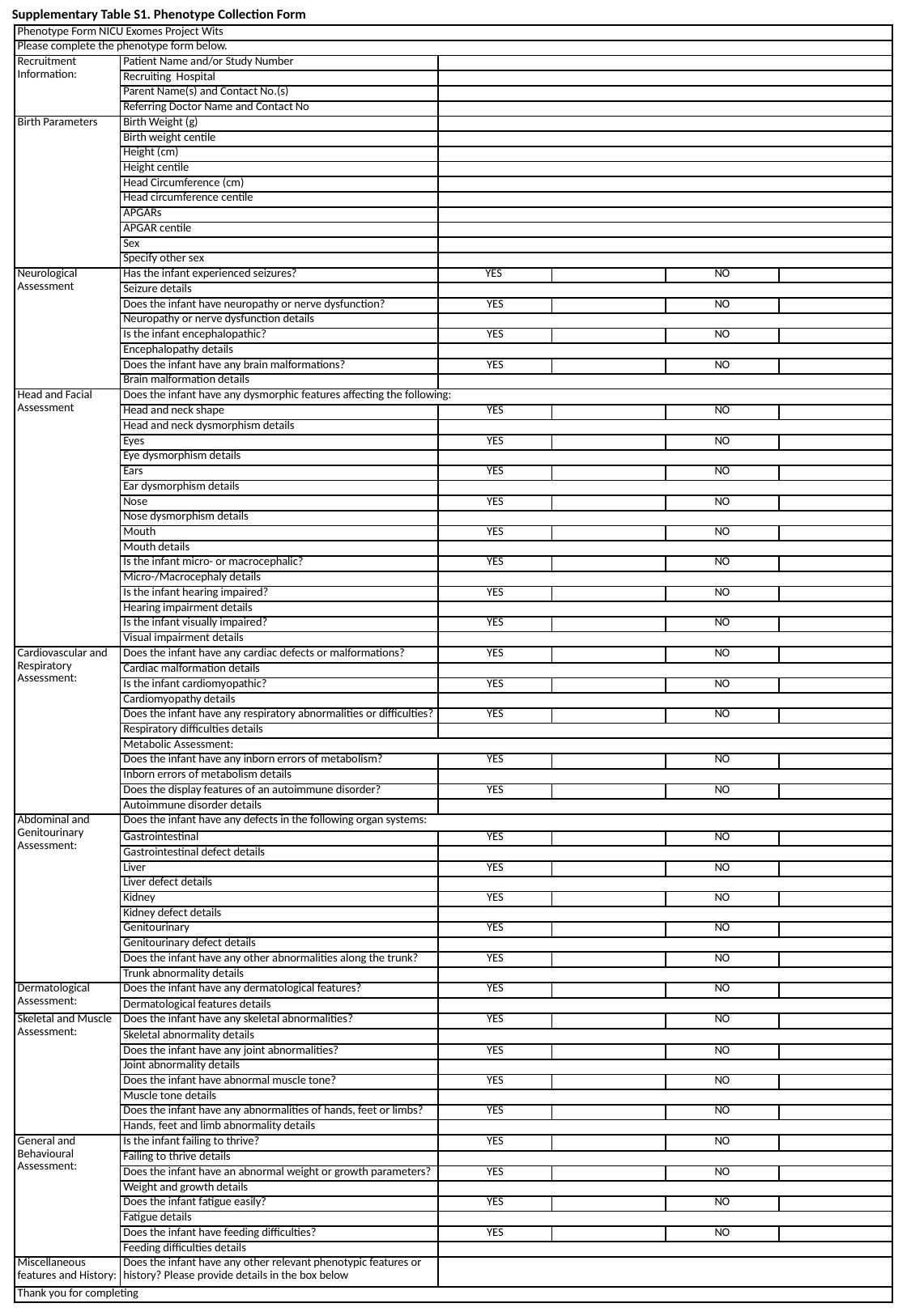

Supplementary Table S1. Phenotype Collection Form
| Phenotype Form NICU Exomes Project Wits | | | | | |
| --- | --- | --- | --- | --- | --- |
| Please complete the phenotype form below. | | | | | |
| Recruitment Information: | Patient Name and/or Study Number | | | | |
| | Recruiting Hospital | | | | |
| | Parent Name(s) and Contact No.(s) | | | | |
| | Referring Doctor Name and Contact No | | | | |
| Birth Parameters | Birth Weight (g) | | | | |
| | Birth weight centile | | | | |
| | Height (cm) | | | | |
| | Height centile | | | | |
| | Head Circumference (cm) | | | | |
| | Head circumference centile | | | | |
| | APGARs | | | | |
| | APGAR centile | | | | |
| | Sex | | | | |
| | Specify other sex | | | | |
| Neurological Assessment | Has the infant experienced seizures? | YES | | NO | |
| | Seizure details | | | | |
| | Does the infant have neuropathy or nerve dysfunction? | YES | | NO | |
| | Neuropathy or nerve dysfunction details | | | | |
| | Is the infant encephalopathic? | YES | | NO | |
| | Encephalopathy details | | | | |
| | Does the infant have any brain malformations? | YES | | NO | |
| | Brain malformation details | | | | |
| Head and Facial Assessment | Does the infant have any dysmorphic features affecting the following: | | | | |
| | Head and neck shape | YES | | NO | |
| | Head and neck dysmorphism details | | | | |
| | Eyes | YES | | NO | |
| | Eye dysmorphism details | | | | |
| | Ears | YES | | NO | |
| | Ear dysmorphism details | | | | |
| | Nose | YES | | NO | |
| | Nose dysmorphism details | | | | |
| | Mouth | YES | | NO | |
| | Mouth details | | | | |
| | Is the infant micro- or macrocephalic? | YES | | NO | |
| | Micro-/Macrocephaly details | | | | |
| | Is the infant hearing impaired? | YES | | NO | |
| | Hearing impairment details | | | | |
| | Is the infant visually impaired? | YES | | NO | |
| | Visual impairment details | | | | |
| Cardiovascular and Respiratory Assessment: | Does the infant have any cardiac defects or malformations? | YES | | NO | |
| | Cardiac malformation details | | | | |
| | Is the infant cardiomyopathic? | YES | | NO | |
| | Cardiomyopathy details | | | | |
| | Does the infant have any respiratory abnormalities or difficulties? | YES | | NO | |
| | Respiratory difficulties details | | | | |
| | Metabolic Assessment: | | | | |
| | Does the infant have any inborn errors of metabolism? | YES | | NO | |
| | Inborn errors of metabolism details | | | | |
| | Does the display features of an autoimmune disorder? | YES | | NO | |
| | Autoimmune disorder details | | | | |
| Abdominal and Genitourinary Assessment: | Does the infant have any defects in the following organ systems: | | | | |
| | Gastrointestinal | YES | | NO | |
| | Gastrointestinal defect details | | | | |
| | Liver | YES | | NO | |
| | Liver defect details | | | | |
| | Kidney | YES | | NO | |
| | Kidney defect details | | | | |
| | Genitourinary | YES | | NO | |
| | Genitourinary defect details | | | | |
| | Does the infant have any other abnormalities along the trunk? | YES | | NO | |
| | Trunk abnormality details | | | | |
| Dermatological Assessment: | Does the infant have any dermatological features? | YES | | NO | |
| | Dermatological features details | | | | |
| Skeletal and Muscle Assessment: | Does the infant have any skeletal abnormalities? | YES | | NO | |
| | Skeletal abnormality details | | | | |
| | Does the infant have any joint abnormalities? | YES | | NO | |
| | Joint abnormality details | | | | |
| | Does the infant have abnormal muscle tone? | YES | | NO | |
| | Muscle tone details | | | | |
| | Does the infant have any abnormalities of hands, feet or limbs? | YES | | NO | |
| | Hands, feet and limb abnormality details | | | | |
| General and Behavioural Assessment: | Is the infant failing to thrive? | YES | | NO | |
| | Failing to thrive details | | | | |
| | Does the infant have an abnormal weight or growth parameters? | YES | | NO | |
| | Weight and growth details | | | | |
| | Does the infant fatigue easily? | YES | | NO | |
| | Fatigue details | | | | |
| | Does the infant have feeding difficulties? | YES | | NO | |
| | Feeding difficulties details | | | | |
| Miscellaneous features and History: | Does the infant have any other relevant phenotypic features or history? Please provide details in the box below | | | | |
| Thank you for completing | | | | | |

## Slide 2
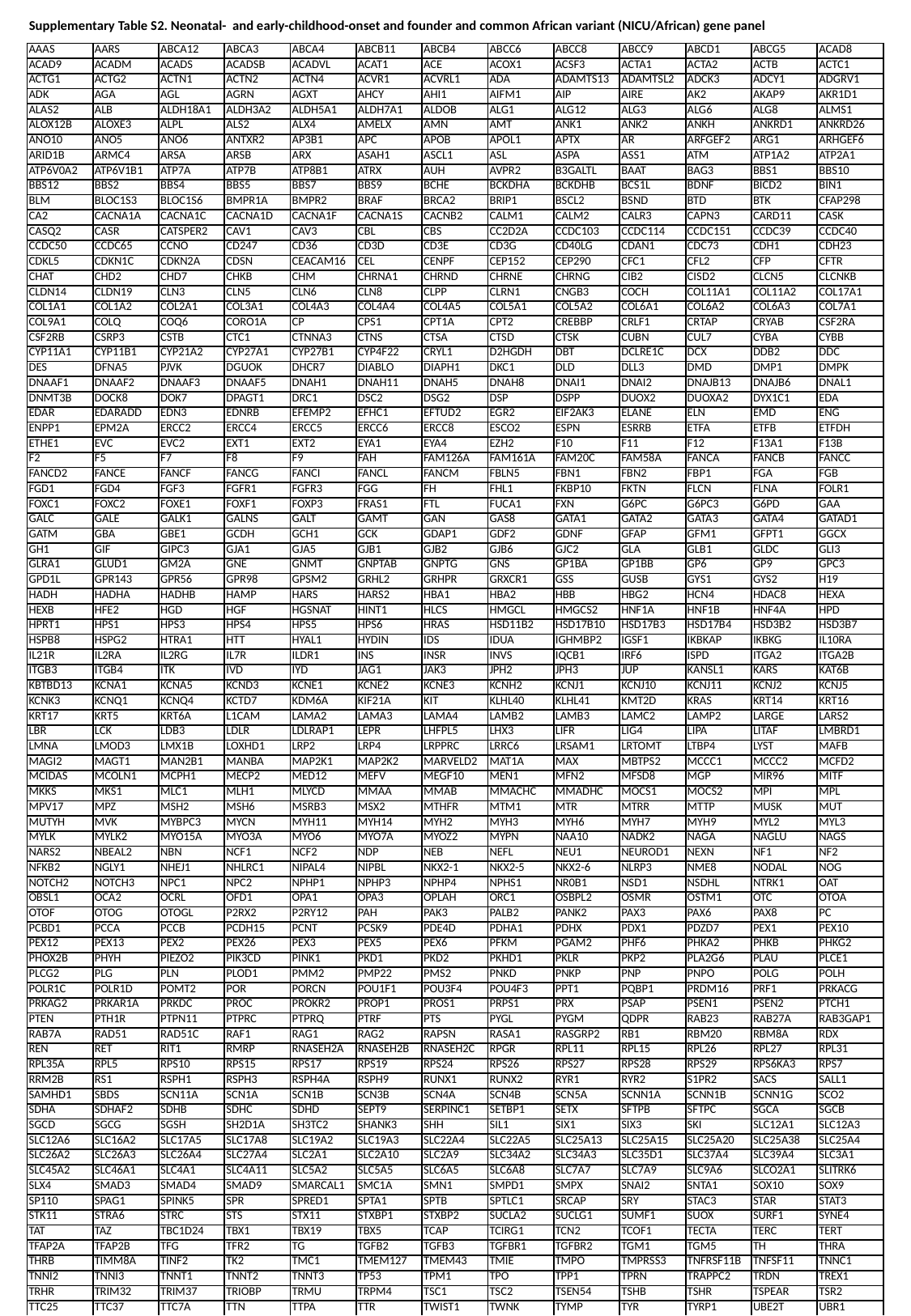

Supplementary Table S2. Neonatal- and early-childhood-onset and founder and common African variant (NICU/African) gene panel
| AAAS | AARS | ABCA12 | ABCA3 | ABCA4 | ABCB11 | ABCB4 | ABCC6 | ABCC8 | ABCC9 | ABCD1 | ABCG5 | ACAD8 |
| --- | --- | --- | --- | --- | --- | --- | --- | --- | --- | --- | --- | --- |
| ACAD9 | ACADM | ACADS | ACADSB | ACADVL | ACAT1 | ACE | ACOX1 | ACSF3 | ACTA1 | ACTA2 | ACTB | ACTC1 |
| ACTG1 | ACTG2 | ACTN1 | ACTN2 | ACTN4 | ACVR1 | ACVRL1 | ADA | ADAMTS13 | ADAMTSL2 | ADCK3 | ADCY1 | ADGRV1 |
| ADK | AGA | AGL | AGRN | AGXT | AHCY | AHI1 | AIFM1 | AIP | AIRE | AK2 | AKAP9 | AKR1D1 |
| ALAS2 | ALB | ALDH18A1 | ALDH3A2 | ALDH5A1 | ALDH7A1 | ALDOB | ALG1 | ALG12 | ALG3 | ALG6 | ALG8 | ALMS1 |
| ALOX12B | ALOXE3 | ALPL | ALS2 | ALX4 | AMELX | AMN | AMT | ANK1 | ANK2 | ANKH | ANKRD1 | ANKRD26 |
| ANO10 | ANO5 | ANO6 | ANTXR2 | AP3B1 | APC | APOB | APOL1 | APTX | AR | ARFGEF2 | ARG1 | ARHGEF6 |
| ARID1B | ARMC4 | ARSA | ARSB | ARX | ASAH1 | ASCL1 | ASL | ASPA | ASS1 | ATM | ATP1A2 | ATP2A1 |
| ATP6V0A2 | ATP6V1B1 | ATP7A | ATP7B | ATP8B1 | ATRX | AUH | AVPR2 | B3GALTL | BAAT | BAG3 | BBS1 | BBS10 |
| BBS12 | BBS2 | BBS4 | BBS5 | BBS7 | BBS9 | BCHE | BCKDHA | BCKDHB | BCS1L | BDNF | BICD2 | BIN1 |
| BLM | BLOC1S3 | BLOC1S6 | BMPR1A | BMPR2 | BRAF | BRCA2 | BRIP1 | BSCL2 | BSND | BTD | BTK | CFAP298 |
| CA2 | CACNA1A | CACNA1C | CACNA1D | CACNA1F | CACNA1S | CACNB2 | CALM1 | CALM2 | CALR3 | CAPN3 | CARD11 | CASK |
| CASQ2 | CASR | CATSPER2 | CAV1 | CAV3 | CBL | CBS | CC2D2A | CCDC103 | CCDC114 | CCDC151 | CCDC39 | CCDC40 |
| CCDC50 | CCDC65 | CCNO | CD247 | CD36 | CD3D | CD3E | CD3G | CD40LG | CDAN1 | CDC73 | CDH1 | CDH23 |
| CDKL5 | CDKN1C | CDKN2A | CDSN | CEACAM16 | CEL | CENPF | CEP152 | CEP290 | CFC1 | CFL2 | CFP | CFTR |
| CHAT | CHD2 | CHD7 | CHKB | CHM | CHRNA1 | CHRND | CHRNE | CHRNG | CIB2 | CISD2 | CLCN5 | CLCNKB |
| CLDN14 | CLDN19 | CLN3 | CLN5 | CLN6 | CLN8 | CLPP | CLRN1 | CNGB3 | COCH | COL11A1 | COL11A2 | COL17A1 |
| COL1A1 | COL1A2 | COL2A1 | COL3A1 | COL4A3 | COL4A4 | COL4A5 | COL5A1 | COL5A2 | COL6A1 | COL6A2 | COL6A3 | COL7A1 |
| COL9A1 | COLQ | COQ6 | CORO1A | CP | CPS1 | CPT1A | CPT2 | CREBBP | CRLF1 | CRTAP | CRYAB | CSF2RA |
| CSF2RB | CSRP3 | CSTB | CTC1 | CTNNA3 | CTNS | CTSA | CTSD | CTSK | CUBN | CUL7 | CYBA | CYBB |
| CYP11A1 | CYP11B1 | CYP21A2 | CYP27A1 | CYP27B1 | CYP4F22 | CRYL1 | D2HGDH | DBT | DCLRE1C | DCX | DDB2 | DDC |
| DES | DFNA5 | PJVK | DGUOK | DHCR7 | DIABLO | DIAPH1 | DKC1 | DLD | DLL3 | DMD | DMP1 | DMPK |
| DNAAF1 | DNAAF2 | DNAAF3 | DNAAF5 | DNAH1 | DNAH11 | DNAH5 | DNAH8 | DNAI1 | DNAI2 | DNAJB13 | DNAJB6 | DNAL1 |
| DNMT3B | DOCK8 | DOK7 | DPAGT1 | DRC1 | DSC2 | DSG2 | DSP | DSPP | DUOX2 | DUOXA2 | DYX1C1 | EDA |
| EDAR | EDARADD | EDN3 | EDNRB | EFEMP2 | EFHC1 | EFTUD2 | EGR2 | EIF2AK3 | ELANE | ELN | EMD | ENG |
| ENPP1 | EPM2A | ERCC2 | ERCC4 | ERCC5 | ERCC6 | ERCC8 | ESCO2 | ESPN | ESRRB | ETFA | ETFB | ETFDH |
| ETHE1 | EVC | EVC2 | EXT1 | EXT2 | EYA1 | EYA4 | EZH2 | F10 | F11 | F12 | F13A1 | F13B |
| F2 | F5 | F7 | F8 | F9 | FAH | FAM126A | FAM161A | FAM20C | FAM58A | FANCA | FANCB | FANCC |
| FANCD2 | FANCE | FANCF | FANCG | FANCI | FANCL | FANCM | FBLN5 | FBN1 | FBN2 | FBP1 | FGA | FGB |
| FGD1 | FGD4 | FGF3 | FGFR1 | FGFR3 | FGG | FH | FHL1 | FKBP10 | FKTN | FLCN | FLNA | FOLR1 |
| FOXC1 | FOXC2 | FOXE1 | FOXF1 | FOXP3 | FRAS1 | FTL | FUCA1 | FXN | G6PC | G6PC3 | G6PD | GAA |
| GALC | GALE | GALK1 | GALNS | GALT | GAMT | GAN | GAS8 | GATA1 | GATA2 | GATA3 | GATA4 | GATAD1 |
| GATM | GBA | GBE1 | GCDH | GCH1 | GCK | GDAP1 | GDF2 | GDNF | GFAP | GFM1 | GFPT1 | GGCX |
| GH1 | GIF | GIPC3 | GJA1 | GJA5 | GJB1 | GJB2 | GJB6 | GJC2 | GLA | GLB1 | GLDC | GLI3 |
| GLRA1 | GLUD1 | GM2A | GNE | GNMT | GNPTAB | GNPTG | GNS | GP1BA | GP1BB | GP6 | GP9 | GPC3 |
| GPD1L | GPR143 | GPR56 | GPR98 | GPSM2 | GRHL2 | GRHPR | GRXCR1 | GSS | GUSB | GYS1 | GYS2 | H19 |
| HADH | HADHA | HADHB | HAMP | HARS | HARS2 | HBA1 | HBA2 | HBB | HBG2 | HCN4 | HDAC8 | HEXA |
| HEXB | HFE2 | HGD | HGF | HGSNAT | HINT1 | HLCS | HMGCL | HMGCS2 | HNF1A | HNF1B | HNF4A | HPD |
| HPRT1 | HPS1 | HPS3 | HPS4 | HPS5 | HPS6 | HRAS | HSD11B2 | HSD17B10 | HSD17B3 | HSD17B4 | HSD3B2 | HSD3B7 |
| HSPB8 | HSPG2 | HTRA1 | HTT | HYAL1 | HYDIN | IDS | IDUA | IGHMBP2 | IGSF1 | IKBKAP | IKBKG | IL10RA |
| IL21R | IL2RA | IL2RG | IL7R | ILDR1 | INS | INSR | INVS | IQCB1 | IRF6 | ISPD | ITGA2 | ITGA2B |
| ITGB3 | ITGB4 | ITK | IVD | IYD | JAG1 | JAK3 | JPH2 | JPH3 | JUP | KANSL1 | KARS | KAT6B |
| KBTBD13 | KCNA1 | KCNA5 | KCND3 | KCNE1 | KCNE2 | KCNE3 | KCNH2 | KCNJ1 | KCNJ10 | KCNJ11 | KCNJ2 | KCNJ5 |
| KCNK3 | KCNQ1 | KCNQ4 | KCTD7 | KDM6A | KIF21A | KIT | KLHL40 | KLHL41 | KMT2D | KRAS | KRT14 | KRT16 |
| KRT17 | KRT5 | KRT6A | L1CAM | LAMA2 | LAMA3 | LAMA4 | LAMB2 | LAMB3 | LAMC2 | LAMP2 | LARGE | LARS2 |
| LBR | LCK | LDB3 | LDLR | LDLRAP1 | LEPR | LHFPL5 | LHX3 | LIFR | LIG4 | LIPA | LITAF | LMBRD1 |
| LMNA | LMOD3 | LMX1B | LOXHD1 | LRP2 | LRP4 | LRPPRC | LRRC6 | LRSAM1 | LRTOMT | LTBP4 | LYST | MAFB |
| MAGI2 | MAGT1 | MAN2B1 | MANBA | MAP2K1 | MAP2K2 | MARVELD2 | MAT1A | MAX | MBTPS2 | MCCC1 | MCCC2 | MCFD2 |
| MCIDAS | MCOLN1 | MCPH1 | MECP2 | MED12 | MEFV | MEGF10 | MEN1 | MFN2 | MFSD8 | MGP | MIR96 | MITF |
| MKKS | MKS1 | MLC1 | MLH1 | MLYCD | MMAA | MMAB | MMACHC | MMADHC | MOCS1 | MOCS2 | MPI | MPL |
| MPV17 | MPZ | MSH2 | MSH6 | MSRB3 | MSX2 | MTHFR | MTM1 | MTR | MTRR | MTTP | MUSK | MUT |
| MUTYH | MVK | MYBPC3 | MYCN | MYH11 | MYH14 | MYH2 | MYH3 | MYH6 | MYH7 | MYH9 | MYL2 | MYL3 |
| MYLK | MYLK2 | MYO15A | MYO3A | MYO6 | MYO7A | MYOZ2 | MYPN | NAA10 | NADK2 | NAGA | NAGLU | NAGS |
| NARS2 | NBEAL2 | NBN | NCF1 | NCF2 | NDP | NEB | NEFL | NEU1 | NEUROD1 | NEXN | NF1 | NF2 |
| NFKB2 | NGLY1 | NHEJ1 | NHLRC1 | NIPAL4 | NIPBL | NKX2-1 | NKX2-5 | NKX2-6 | NLRP3 | NME8 | NODAL | NOG |
| NOTCH2 | NOTCH3 | NPC1 | NPC2 | NPHP1 | NPHP3 | NPHP4 | NPHS1 | NR0B1 | NSD1 | NSDHL | NTRK1 | OAT |
| OBSL1 | OCA2 | OCRL | OFD1 | OPA1 | OPA3 | OPLAH | ORC1 | OSBPL2 | OSMR | OSTM1 | OTC | OTOA |
| OTOF | OTOG | OTOGL | P2RX2 | P2RY12 | PAH | PAK3 | PALB2 | PANK2 | PAX3 | PAX6 | PAX8 | PC |
| PCBD1 | PCCA | PCCB | PCDH15 | PCNT | PCSK9 | PDE4D | PDHA1 | PDHX | PDX1 | PDZD7 | PEX1 | PEX10 |
| PEX12 | PEX13 | PEX2 | PEX26 | PEX3 | PEX5 | PEX6 | PFKM | PGAM2 | PHF6 | PHKA2 | PHKB | PHKG2 |
| PHOX2B | PHYH | PIEZO2 | PIK3CD | PINK1 | PKD1 | PKD2 | PKHD1 | PKLR | PKP2 | PLA2G6 | PLAU | PLCE1 |
| PLCG2 | PLG | PLN | PLOD1 | PMM2 | PMP22 | PMS2 | PNKD | PNKP | PNP | PNPO | POLG | POLH |
| POLR1C | POLR1D | POMT2 | POR | PORCN | POU1F1 | POU3F4 | POU4F3 | PPT1 | PQBP1 | PRDM16 | PRF1 | PRKACG |
| PRKAG2 | PRKAR1A | PRKDC | PROC | PROKR2 | PROP1 | PROS1 | PRPS1 | PRX | PSAP | PSEN1 | PSEN2 | PTCH1 |
| PTEN | PTH1R | PTPN11 | PTPRC | PTPRQ | PTRF | PTS | PYGL | PYGM | QDPR | RAB23 | RAB27A | RAB3GAP1 |
| RAB7A | RAD51 | RAD51C | RAF1 | RAG1 | RAG2 | RAPSN | RASA1 | RASGRP2 | RB1 | RBM20 | RBM8A | RDX |
| REN | RET | RIT1 | RMRP | RNASEH2A | RNASEH2B | RNASEH2C | RPGR | RPL11 | RPL15 | RPL26 | RPL27 | RPL31 |
| RPL35A | RPL5 | RPS10 | RPS15 | RPS17 | RPS19 | RPS24 | RPS26 | RPS27 | RPS28 | RPS29 | RPS6KA3 | RPS7 |
| RRM2B | RS1 | RSPH1 | RSPH3 | RSPH4A | RSPH9 | RUNX1 | RUNX2 | RYR1 | RYR2 | S1PR2 | SACS | SALL1 |
| SAMHD1 | SBDS | SCN11A | SCN1A | SCN1B | SCN3B | SCN4A | SCN4B | SCN5A | SCNN1A | SCNN1B | SCNN1G | SCO2 |
| SDHA | SDHAF2 | SDHB | SDHC | SDHD | SEPT9 | SERPINC1 | SETBP1 | SETX | SFTPB | SFTPC | SGCA | SGCB |
| SGCD | SGCG | SGSH | SH2D1A | SH3TC2 | SHANK3 | SHH | SIL1 | SIX1 | SIX3 | SKI | SLC12A1 | SLC12A3 |
| SLC12A6 | SLC16A2 | SLC17A5 | SLC17A8 | SLC19A2 | SLC19A3 | SLC22A4 | SLC22A5 | SLC25A13 | SLC25A15 | SLC25A20 | SLC25A38 | SLC25A4 |
| SLC26A2 | SLC26A3 | SLC26A4 | SLC27A4 | SLC2A1 | SLC2A10 | SLC2A9 | SLC34A2 | SLC34A3 | SLC35D1 | SLC37A4 | SLC39A4 | SLC3A1 |
| SLC45A2 | SLC46A1 | SLC4A1 | SLC4A11 | SLC5A2 | SLC5A5 | SLC6A5 | SLC6A8 | SLC7A7 | SLC7A9 | SLC9A6 | SLCO2A1 | SLITRK6 |
| SLX4 | SMAD3 | SMAD4 | SMAD9 | SMARCAL1 | SMC1A | SMN1 | SMPD1 | SMPX | SNAI2 | SNTA1 | SOX10 | SOX9 |
| SP110 | SPAG1 | SPINK5 | SPR | SPRED1 | SPTA1 | SPTB | SPTLC1 | SRCAP | SRY | STAC3 | STAR | STAT3 |
| STK11 | STRA6 | STRC | STS | STX11 | STXBP1 | STXBP2 | SUCLA2 | SUCLG1 | SUMF1 | SUOX | SURF1 | SYNE4 |
| TAT | TAZ | TBC1D24 | TBX1 | TBX19 | TBX5 | TCAP | TCIRG1 | TCN2 | TCOF1 | TECTA | TERC | TERT |
| TFAP2A | TFAP2B | TFG | TFR2 | TG | TGFB2 | TGFB3 | TGFBR1 | TGFBR2 | TGM1 | TGM5 | TH | THRA |
| THRB | TIMM8A | TINF2 | TK2 | TMC1 | TMEM127 | TMEM43 | TMIE | TMPO | TMPRSS3 | TNFRSF11B | TNFSF11 | TNNC1 |
| TNNI2 | TNNI3 | TNNT1 | TNNT2 | TNNT3 | TP53 | TPM1 | TPO | TPP1 | TPRN | TRAPPC2 | TRDN | TREX1 |
| TRHR | TRIM32 | TRIM37 | TRIOBP | TRMU | TRPM4 | TSC1 | TSC2 | TSEN54 | TSHB | TSHR | TSPEAR | TSR2 |
| TTC25 | TTC37 | TTC7A | TTN | TTPA | TTR | TWIST1 | TWNK | TYMP | TYR | TYRP1 | UBE2T | UBR1 |
| UGT1A1 | UMOD | UNC13D | UROD | UROS | USH1C | USH1G | USH2A | VCAN | VCL | VCP | VDR | VHL |
| VIPAS39 | VLDLR | VPS13A | VPS13B | VPS33B | VWF | WAS | WDR62 | WFS1 | WHRN | WNT10A | WRN | WT1 |
| XPA | XPC | ZAP70 | ZEB2 | ZIC2 | ZIC3 | ZMPSTE24 | ZMYND10 | ZNF469 | | | | |
